# Chaperone-mediated autophagy as a modulator of aging and longevity

**DOI:** 10.3389/fragi.2024.1509400

**Published:** 2024-12-02

**Authors:** S. Joseph Endicott

**Affiliations:** ^1^ Department of Pathology, University of New Mexico Health Sciences Center, Albuquerque, NM, United States; ^2^ Autophagy, Inflammation, and Metabolism Center of Biomedical Research Excellence, (AIM CoBRE), University of New Mexico Health Sciences Center, Albuquerque, NM, United States

**Keywords:** aging, autophagy, chaperone-mediated autophagy, longevity, metabolism

## Abstract

Chaperone-mediated autophagy (CMA) is the lysosomal degradation of individually selected proteins, independent of vesicle fusion. CMA is a central part of the proteostasis network in vertebrate cells. However, CMA is also a negative regulator of anabolism, and it degrades enzymes required for glycolysis, *de novo* lipogenesis, and translation at the cytoplasmic ribosome. Recently, CMA has gained attention as a possible modulator of rodent aging. Two mechanistic models have been proposed to explain the relationship between CMA and aging in mice. Both of these models are backed by experimental data, and they are not mutually exclusionary. Model 1, the “Longevity Model,” states that lifespan-extending interventions that decrease signaling through the INS/IGF1 signaling axis also increase CMA, which degrades (and thereby reduces the abundance of) several proteins that negatively regulate vertebrate lifespan, such as MYC, NLRP3, ACLY, and ACSS2. Therefore, enhanced CMA, in early and midlife, is hypothesized to slow the aging process. Model 2, the “Aging Model,” states that changes in lysosomal membrane dynamics with age lead to age-related losses in the essential CMA component LAMP2A, which in turn reduces CMA, contributes to age-related proteostasis collapse, and leads to overaccumulation of proteins that contribute to age-related diseases, such as Alzheimer’s disease, Parkinson’s disease, cancer, atherosclerosis, and sterile inflammation. The objective of this review paper is to comprehensively describe the data in support of both of these explanatory models, and to discuss the strengths and limitations of each.

## 1 Introduction

Chaperone-mediated autophagy (CMA) is a highly selective form of lysosomal proteolysis, where proteins bearing consensus motifs are individually selected for lysosomal degradation (Dice, 1990; Cuervo and Dice, 1996; Cuervo et al., 1997). CMA is mechanistically distinct from macroautophagy and microautophagy, which, along with CMA, are present in most mammalian cells types.

**FIGURE 1:**
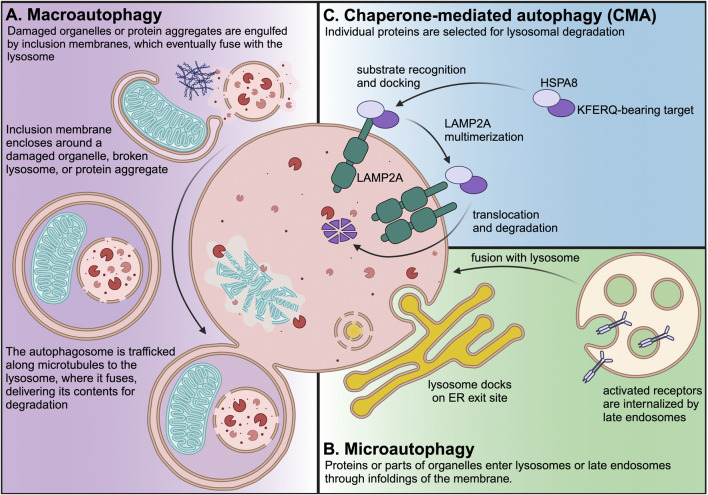
Three branches of autophagy are present in most mammalian cell types. Macroautophagy is the most well characterized branch of autophagy, and it is easily recognized by the morphology of the phagophores, autophagosomes, and lysosomes. Macroautophagy can be non-selective, but it also selectively targets damaged organelles or protein aggregates, which are engulfed by double-membrane phagophores, which seal to form autophagosomes. Autophagosomes are trafficked along the microtubule cytoskeleton to lysosomes, where they fuse to deliver their contents for degradation. Microautophagy is an ESCRT-dependent process that occurs at lysosome contact sites, such as ER exit sites. Endosomal microautophagy occurs at the membrane of the late endosome/multivesicular body to degrade endocytosed receptors or proteins carried to endosomes by cytosolic chaperones. Papers with “Microautophagy” in the title represent <0.25% of PubMed-indexed papers with “autophagy” in the title. CMA is the lysosomal degradation of individually selected proteins that contain KFERQ-like consensus motifs. CMA requires HSPA8 (which recognizes the KFERQ motif in CMA target proteins) and LAMP2A, which is the core component of the translocation complex that carries CMA target proteins into the lysosomal lumen. Papers with “Chaperone-mediated autophagy” in the title represent nearly 1% of PubMed-indexed papers with “autophagy” in the title.

Macroautophagy (Figure 1A) begins when inclusion membranes (phagophores) engulf large swaths of cytoplasm or organelles, and then seal to form double-membrane autophagosomes. Autophagosomes then fuse with lysosomes, delivering their contents for degradation by lysosomal hydrolases (Galluzzi et al., 2017). Macroautophagy was the first branch of autophagy to be discovered, and it is easily recognized in electron micrograms, based on the morphology of phagophores, autophagosomes, and lysosomes (Galluzzi et al., 2017).

Microautophagy (Figure 1B) can occur at the lysosome or the late endosome when part of the membrane folds inward and pinches off into the lumen. Endosomal microautophagy of cytosolic proteins is selective, and requires HSPA8 both for cargo recognition and for deforming the membrane as part of cargo internalization (Sahu et al., 2011; Uytterhoeven et al., 2015). Microautophagy can also occur directly at the lysosomal membrane. For example, in response to nutrient stress, lysosomes can dock onto endoplasmic reticulum (ER) exit sites and engulf COPII vesicles (Liao et al., 2024).

CMA (Figure 1C) does not require vesicle fusion or microtubule-based vesicle trafficking (Finn et al., 2005; Kaushik and Cuervo, 2018). Rather, CMA target proteins are translocated across the lysosomal membrane in a manner that requires the lysosomal transmembrane protein LAMP2A (Cuervo and Dice, 1996). LAMP2A is the protein product of one of three splice variants of the *Lamp2* mRNA. All *Lamp2* variants share the first eight exons. However, *Lamp2a* mRNA differs from the *Lamp2b* and *Lamp2c* variants by the ninth and final exon, which encodes the transmembrane domain and cytoplasmic tail (Eskelinen et al., 2005). Genetic depletion of LAMP2A (but not the other LAMP2 variants) by targeting the unique ninth exon is considered to be the most selective way of blocking CMA (Kaushik and Cuervo, 2018).

Proteins targeted for degradation by CMA contain a consensus motif resembling the amino acid sequence KFERQ, which is recognized by the cytosolic chaperone HSPA8 (also known as HSC70 or HSC73) (Chiang et al., 1989; Dice, 1990; Agarraberes et al., 1997). HSPA8, along with other cytosolic chaperones, participates in the trafficking of the KFERQ-bearing target to the lysosomal surface, where it is translocated across the lysosomal membrane by a complex containing LAMP2A (Chiang et al., 1989; Cuervo and Dice, 1996; Agarraberes et al., 1997; Bandyopadhyay et al., 2008).

The KFERQ motif is not recognized as an exact sequence, but rather by the relative charges of amino acid side chains. The first or fifth amino acid of the pentapeptide motif is glutamine. The remaining four amino acids must contain one or two positive, one negative, and one or two hydrophobic side chains, in any order (Dice, 1990; Majeski and Dice, 2004). The KFERQ motif can be activated or deactivated through addition or removal of post-translational modifications, such as phosphorylation or acetylation, which alter side-chain charges or hydrophobicity (Kirchner et al., 2019). In silico analysis of the mouse proteome suggests that 47% of mouse proteins contain at least one “canonical” KFERQ motif, i.e., a motif that does not require a post-translational modification to be active (Kirchner et al., 2019). When including motifs hypothetically activated by post-translational modifications, 78% of mouse proteins contain at least one KFERQ motif (Kirchner et al., 2019). However, because sequestration of hydrophobic amino acids away from water in the cytosol is a key step in protein folding (Dyson et al., 2006), it is unclear how many KFERQ motifs will be exposed on the protein surface to interact with cytosolic HSPA8. Thus, empirical analysis of protein degradation is required to determine a protein’s status as a CMA target.

Initial studies focused on the role of CMA in proteostasis (Massey et al., 2006; Schneider et al., 2015). Loss of CMA disrupts cellular proteostasis mechanisms, causes an intracellular accumulation of oxidized proteins, and causes a compensatory increase in protein degradation through the proteasome and macroautophagy (Massey et al., 2006; Schneider et al., 2015). However, recent studies have shown that CMA plays a diverse array of functions in mammalian cellular biology, such as regulating the differentiation of adipocytes (Kaushik et al., 2022), and regulating the activation of hematopoietic stem cells (Dong et al., 2021).

The most critical function of CMA might be the selective degradation of a subset of the proteome to downregulate fundamental anabolic processes, including glycolysis, fatty acid synthesis, and translation at the cytoplasmic ribosome (Schneider et al., 2014; Xia et al., 2015; Hao et al., 2019; Kacal et al., 2021; Endicott et al., 2022; Zhang et al., 2023a; Endicott and Miller, 2024). CMA also regulates the abundance of proteins whose overaccumulation contributes to age-related diseases, including Parkinson’s disease, Alzheimer’s disease, fatty-liver disease, oncogenesis, and atherosclerosis (Schneider et al., 2014; Xilouri et al., 2016; Bourdenx et al., 2021; Qiao et al., 2021; Gomez-Sintes et al., 2022).

**FIGURE 2:**
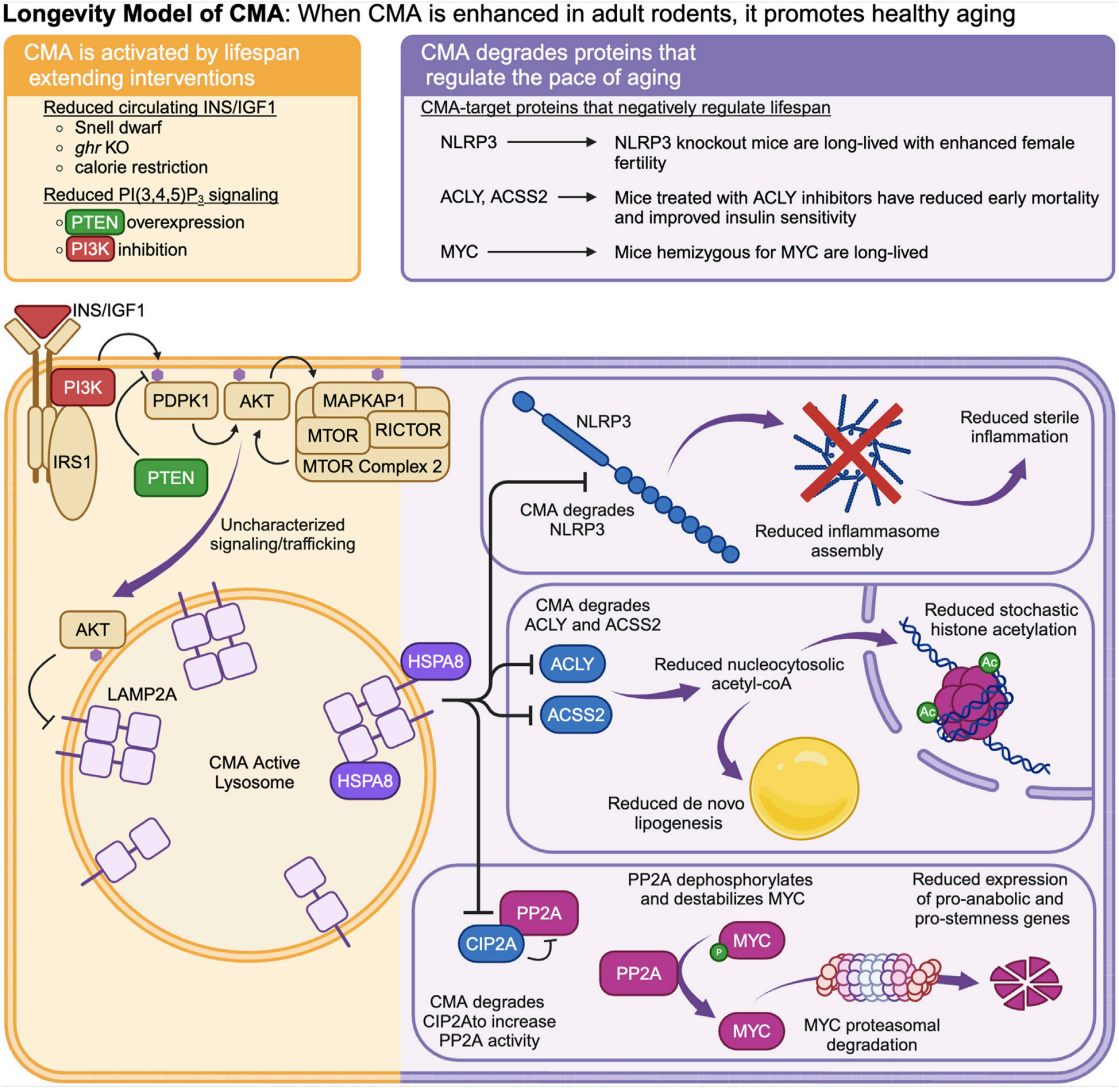
The “Longevity Model” states that when CMA is enhanced in healthy adult animals, it promotes longevity by degrading proteins that promote fast aging. CMA is negatively regulated by intracellular signaling events downstream of the INS/IGF1 signaling cascade. Multiple interventions that extend lifespan in mice reduce signaling through the INS/IGF1 cascade and enhance CMA. CMA degrades and reduces the abundance of MYC, NLRP3, ACLY, and ACSS2.

Recently, there has been a rapid expansion in the volume of literature evaluating the relationship between CMA, aging, and longevity. Two hypothetical models, which are not mutually exclusionary, have emerged to explain the relationship between aging and CMA:

**The Longevity Model** (Figure 2) was first proposed by Endicott and Miller (Endicott and Miller, 2024). This model states that CMA is negatively regulated by intracellular signaling events downstream of the insulin (INS)/insulin-like growth factor 1 (IGF1) signaling axis (Endicott et al., 2020; Endicott et al., 2021; Zhang et al., 2023a). Multiple interventions that extend lifespan in mice reduce INS/IGF1 signaling and enhance CMA (Endicott et al., 2021; Zhang et al., 2023a; Jafari et al., 2024). CMA, in turn, degrades (and reduces the abundance of) proteins that are negative regulators of lifespan, such as MYC (Hofmann et al., 2015), NLRP3 (Marín-Aguilar et al., 2020; Navarro-Pando et al., 2021), ACLY (Peleg et al., 2016), and ACSS2 (Eisenberg et al., 2014). By degrading these negative regulators of lifespan, CMA is responsible for at least some of proteomic and metabolic changes that boost longevity downstream of reduced signaling through the INS/IGF1 axis (Endicott and Miller, 2024).

**FIGURE 3:**
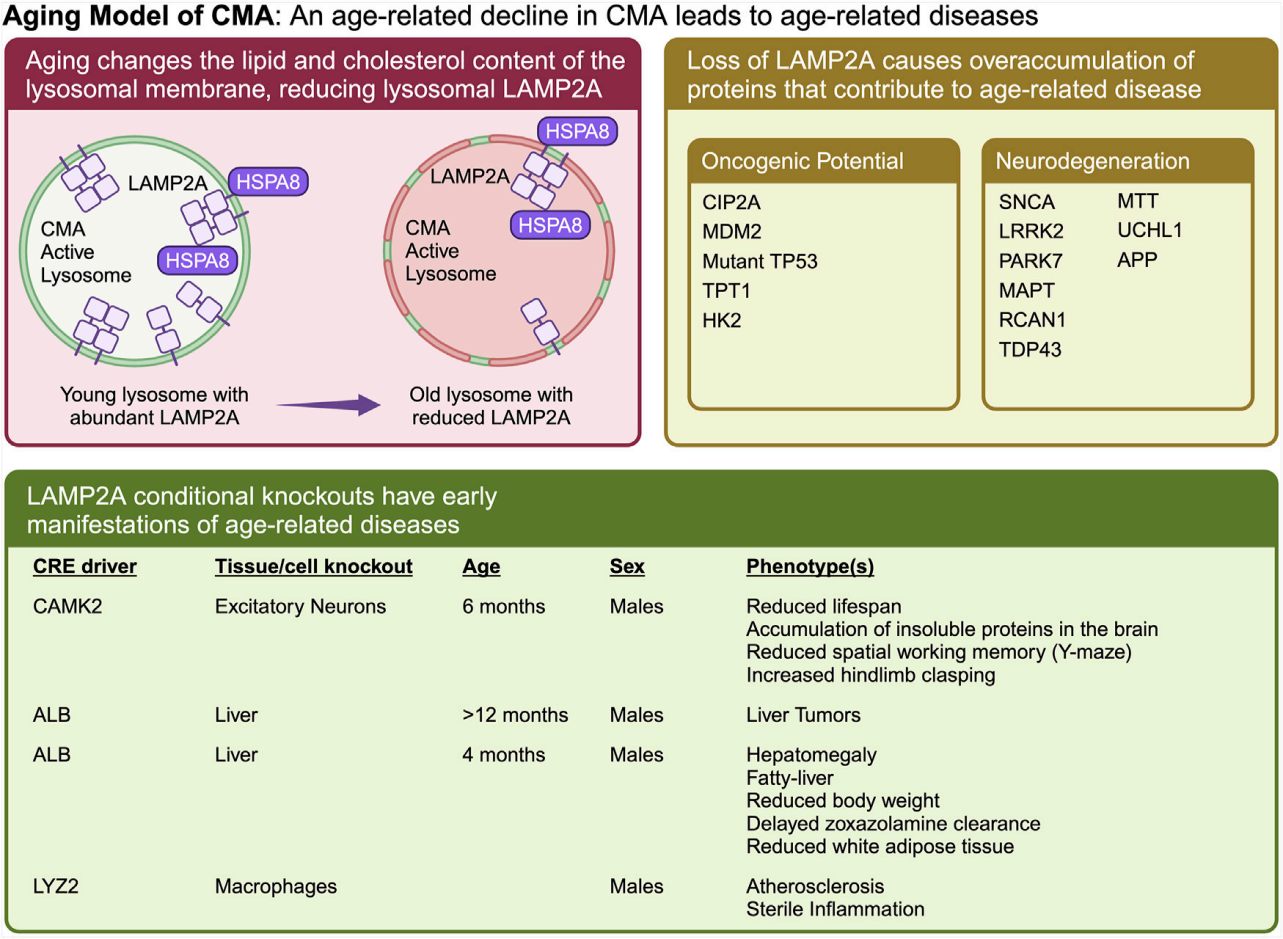
The “Aging Model” states that CMA decreases as a function of age, which leads to an overaccumulation of proteins that cause age-related diseases. Aging causes a change in lysosomal membrane composition, which reduces the stability of LAMP2A at the lysosomal membrane. A reduction in lysosomal LAMP2A with age, leads to reduced CMA, and increased abundance of proteins whose overaccumulation leads to Alzheimer’s, Parkinson’s, cancer, fatty liver disease, and atherosclerosis. Multiple proteins, whose overaccumulation leads to age-related disease, have been identified as CMA targets. Ablation of CMA in the brain or liver, causes neurodegeneration and cancer, respectively.

**The Aging Model** (Figure 3) emerged primarily from the work of Cuervo’s lab, and has been extensively reviewed (Cuervo and Wong, 2014; Tasset and Cuervo, 2016; Kaushik and Cuervo, 2018). This model states that CMA decreases as a function of age (Cuervo and Dice, 2000), due to a reduction in the stability of LAMP2A at the lysosomal membrane with age-related changes in lysosomal lipid composition (Kiffin et al., 2007). The age-related decline in CMA leads to increased abundance of proteins whose overaccumulation leads to Alzheimer’s, Parkinson’s, cancer, fatty liver disease, and atherosclerosis. Restoration of CMA in old animals will delay or reverse age-related proteomic and metabolic changes (Zhang and Cuervo, 2008), and protect against the development of neurodegenerative phenotypes (Bourdenx et al., 2021).

The objective of this review paper is to present the experimental evidence that is relevant to these two models for the role of CMA in aging and longevity. We will begin by describing a few key methods and methodological considerations for evaluating CMA in long-lived or aging rodents. Then, we comprehensively review all relevant literature that (1) identifies genetic and pharmacological interventions that both extend rodent lifespan and activate CMA, (2) describes CMA target proteins that are involved in regulating lifespan, (3) describes changes with CMA as a function of age, and (4) characterizes the pathogenesis of diseases that result from loss of CMA. We discuss the strengths and limitations of both models that explain the relationship between CMA, aging, and longevity.

## 2 Key methods and methodological considerations

Dozens of assays can be used to evaluate CMA (Kaushik and Cuervo, 2009; Patel and Cuervo, 2015). However, in the domain of study where CMA research overlaps with aging research, there are three assays commonly used to evaluate CMA that must be understood.

### 2.1 KFERQ-Dendra2 reporter

**FIGURE 4:**
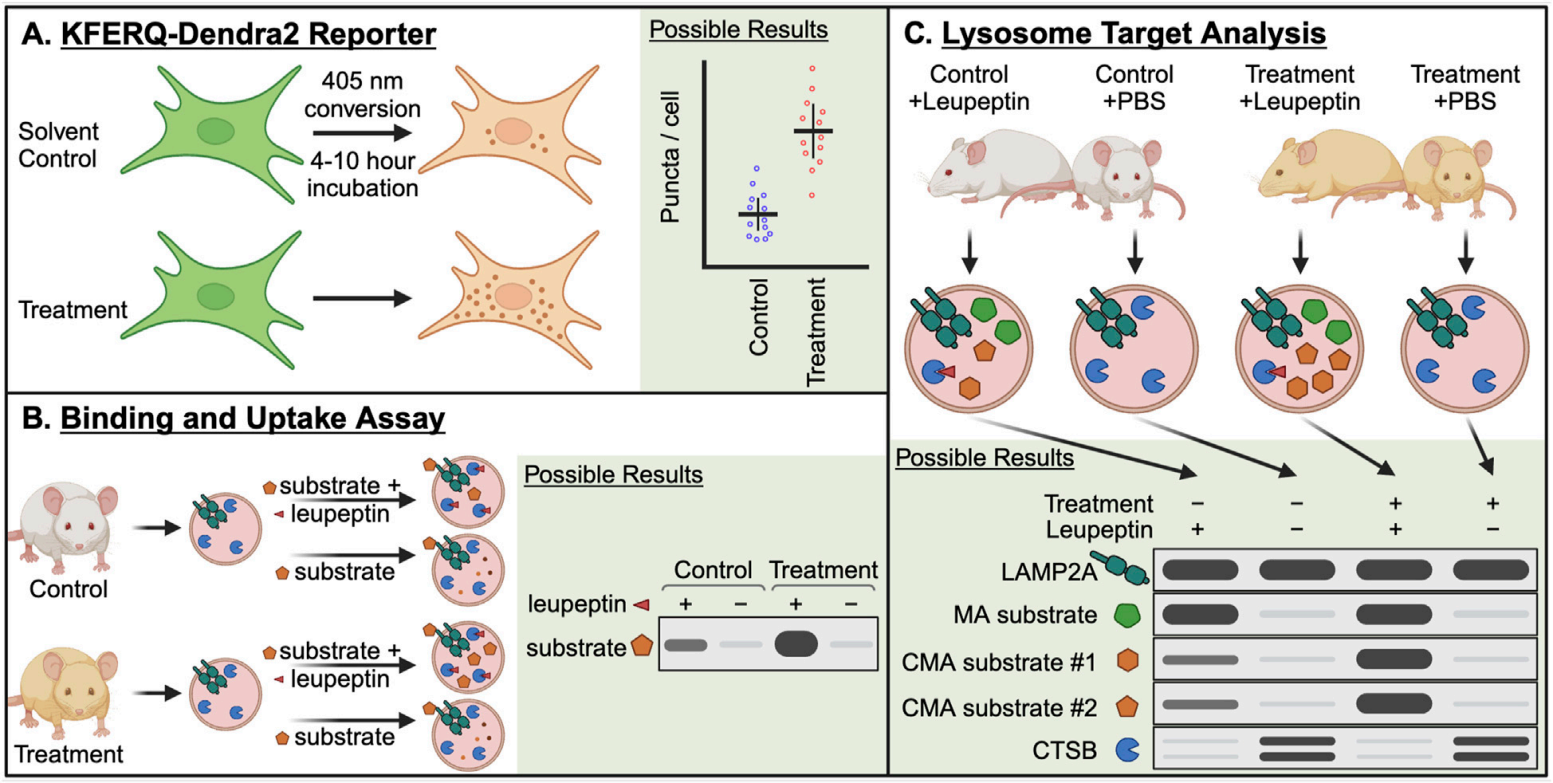
Essential methods for studying CMA in the contexts of aging and longevity. **(A)** The KFERQ-Dendra2 reporter consists of the CMA targeting sequence from RNase A (KFERQ) fused to the photo-switchable protein Dendra2. When CMA is active, this protein accumulates at the lysosome, forming puncta that can be counted to approximate the number of CMA-active lysosomes in a cell. Photo-switching the Dendra2 reduces background noise from newly synthesized protein. **(B)** The binding and uptake assay is the oldest method for studying CMA, and it has several variations. In the simplest variation, lysosomes are isolated from a control and treatment group, which can be mouse tissues or cultured cells. The isolated lysosomes are split into two groups, which are mixed with isotonic buffer containing a recombinant CMA target protein, with or without protease inhibitors. After a brief incubation, the lysosomes are pelleted, washed, and then lysed to measure the accumulation of the CMA target protein by Western blotting. The difference in the target between the protease treated and untreated groups is the amount of protein “uptake” into the lysosomal lumen. **(C)** Lysosome target analysis is used to identify lysosomal uptake of endogenous target proteins. This method requires a minimum of four treatment groups: Control + Leupeptin, Control + PBS, Treatment + Leupeptin, Treatment + PBS. Leupeptin blocks lysosomal proteolysis, without blocking the delivery of proteins to the lysosome. Thus, proteins destined for lysosomal degradation accumulate in the lysosomal lumen. By performing western blots for *bona fide* CMA target proteins, the relative amounts of CMA between the Control and Treatment groups can be approximated.

CMA can be monitored in cell-based systems using a fluorescent reporter, consisting of the N-terminus of RNase A fused to photoswitchable protein Dendra2 (Koga et al., 2011) (Figure 4A). This N-terminal portion of RNase A contains the KFERQ-like targeting motif, which is responsible for targeting the protein for degradation by CMA (Dice et al., 1986; Dice, 1990). This protein accumulates on lysosomes when CMA is active, forming countable puncta, which serve as an estimate for the number of lysosomes actively engaged in CMA in a cell. By photoswitching the Dendra from green to red, and later imaging the cells in the red channel, background fluorescence from newly synthesized proteins (green) is eliminated, allowing for a stronger signal to noise ratio for (red) Dendra proteins sequestered to lysosomes (Koga et al., 2011). Photoswitching is not required for this assay, and in some contexts, it is not possible. For example, photoswitching cannot be achieved in organs of transgenic mice expressing KFERQ-Dendra2, but the presence of puncta in fixed tissue slices can still be used to estimate CMA activity in different cell types (Dong et al., 2020).

### 2.2 Binding and uptake assay

The first assay that could disambiguate CMA activity from other pathways of lysosomal protein degradation was the “binding and uptake assay” (Figure 4B). This assay is based on the finding that lysosome enriched fractions from rat or mouse livers are able to translocate some recombinant soluble proteins into lysosomal lumens (Cuervo et al., 1994). While there are multiple variations of this assay, we have presented the simplest version. At a minimum, this assay requires isolated lysosomes from a control mouse liver or cell population and those from a treated mouse or cell population. The lysosomes from each treatment group are split into two assay groups, where the first group is mixed with a purified recombinant protein that is known to be a CMA target, such as GAPDH, MAPT, or RNase A, and the second group is mixed with the same target protein plus protease inhibitors. After an incubation step in physiological conditions, the lysosomes are washed and resuspended. The lysosomes are lysed and subjected to immunoblotting. The amount of target protein that is recovered in the group without protease inhibitor is subtracted from the amount of target protein that is recovered in the group treated with protease inhibitor. The remaining value is the amount of protein “uptake” into the lysosomal lumen. Differences in uptake between the control group and the treatment group indicate the effect of the treatment on CMA. For additional explanation of these assays, refer to Kaushik and Cuervo (2009) and Patel and Cuervo (2015).

### 2.3 Lysosome target analysis

Lysosome target analysis (Figure 4C) is used to identify the uptake of endogenous proteins into lysosomes, including endogenous CMA targets. This method is most commonly performed in mice (Schneider et al., 2014; Endicott et al., 2020; Endicott et al., 2021; Endicott et al., 2022; Zhang et al., 2023a). However, this method can also be applied to lysosomes isolated from cultured cells (Hao et al., 2019; Kacal et al., 2021). This method requires a minimum of four treatment groups: Control + Leupeptin, Control + PBS (solvent for leupeptin), Treatment + Leupeptin, Treatment + PBS. In the context of this example, “Control” means a genetically normal animal that has not been treated with any CMA activating intervention, and “Treatment” is an animal with a genetic, dietary, or pharmacological intervention that is hypothesized to boost CMA activity. Leupeptin (or PBS solvent control) is injected intraperitoneally into the mice at least 2 h prior to humane euthanasia and isolation of liver lysosomes. Leupeptin is a protease inhibitor that blocks lysosomal proteolysis, without blocking the delivery of proteins to the lysosome. Thus, proteins destined for lysosomal degradation accumulate in the lysosomal lumen. By performing western blots for *bona fide* CMA target proteins, the relative amounts of CMA between the Control and Treatment groups can be approximated. Papers that have used variations of this assay include refs (Schneider et al., 2014; Hao et al., 2019; Kacal et al., 2021; Endicott et al., 2022; Zhang et al., 2023a).

## 3 CMA is enhanced in long-lived mice with reduced PI3K/AKT/MTOR signaling

**FIGURE 5:**
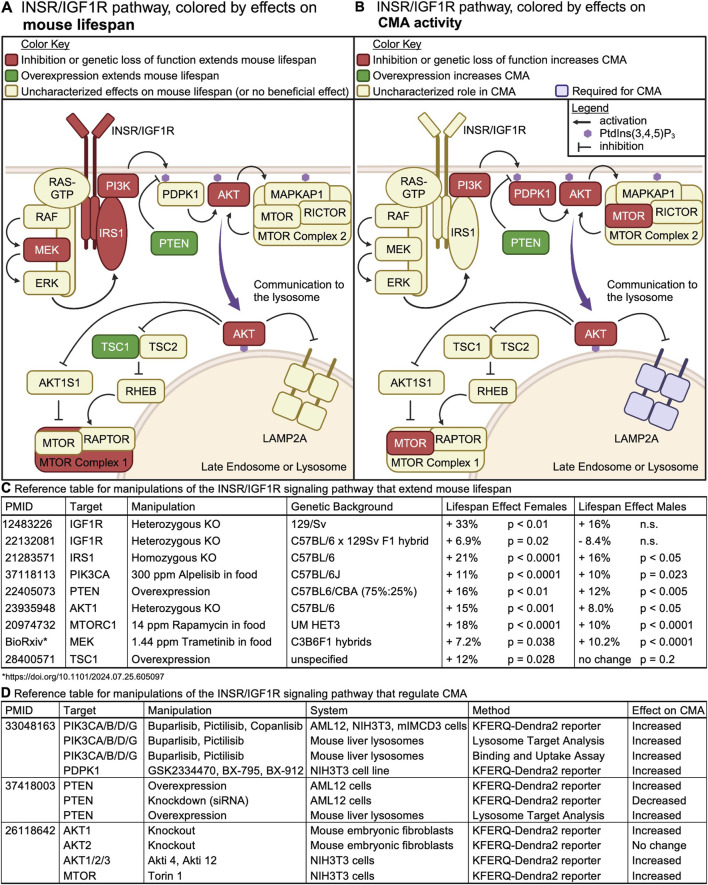
The INS/IGF1 signaling cascade regulates mouse lifespan and CMA. **(A)** Intracellular signaling events downstream of INSR or IGF1R are color-coded by their effects on mouse lifespan. **(B)** Intracellular signaling events downstream of INSR or IGF1R are color-coded by their effects on CMA activity. **(C)** A table containing the reference for information depicted in **(A)**, along with details on the nature of the genetic or pharmacological manipulation, and its effect on lifespan in the two sexes. **(D)** A table containing the reference for information depicted in **(B)**, along with details on the nature of the genetic or pharmacological manipulation, and its effect on CMA.

Downstream of the activation of receptor tyrosine kinases (RTKs), such as the insulin receptor (INSR), class I PI3Ks phosphorylate the phosphatidyl inositol (PI) lipid second messenger PI(4,5)P_2_, producing PI(3,4,5)P_3_. AKT, PDPK1, and MTOR complex 2 (MTORC2) bind to PI(3,4,5)P_3_, bringing them into close proximity to each other (Figure 5A). This allows both PDPK1 and MTORC2 to phosphorylate AKT, leading to the complete activation of AKT’s kinase activity (Bayascas et al., 2008; Liu et al., 2015). Through the phosphorylation of its substrates, AKT regulates several fundamental cellular processes, including growth, survival, glucose uptake, and metabolism (Sugiyama et al., 2019). Furthermore, AKT phosphorylates both AKT1S1 (PRAS40) and TSC2, to relieve their inhibitory effects on MTORC1, thereby boosting MTORC1 activity (Glaviano et al., 2023).

Interventions that reduce signaling through the PI3K/AKT/MTOR pathway reliably extend lifespan in mice, with information about each information summarized in Figure 5C. Figure 5A shows a diagram of the PI3K/AKT/MTOR signaling network, colored by perturbations that increase mouse lifespan.

### 3.1 CMA and mouse aging are both regulated by the PI3K/AKT/MTOR signaling axis

The involvement of MTORC2 and AKT in CMA regulation was first observed by Arias et al. (2015), (Figures 5B, D), who performed a genetic screen that revealed that knockdown of the phosphatase PHLPP1 reduces CMA activity. PHLPP1 dephosphorylates, and thus deactivates, AKT (Arias et al., 2015). AKT is a critical negative regulator of CMA (Arias et al., 2015; Endicott et al., 2020; Zhang et al., 2023a). By assessing CMA in mouse embryonic fibroblasts with deletions of individual AKT orthologs, it was determined that genetic deletion of *akt1* results in constitutive activation of CMA. However, deletion of *akt2* is not sufficient to activate CMA (Arias et al., 2015). Treatment of isolated rat liver lysosomes with AKT inhibitors promotes the multimerization of LAMP2A and enhances uptake of CMA substrates (Arias et al., 2015). Similarly, treatment of isolated rat liver lysosomes with Torin, an MTOR inhibitor, activates CMA. The effects of Torin on CMA are attributed to the inhibition of MTOR complex 2 (MTORC2) (Arias et al., 2015), because MTORC2 increases AKT activity by phosphorylating it on S473. Thus, AKT controls CMA through direct effects on the lysosomal membrane.

Endicott et al. (2020) built upon this initial work by showing that class I PI3Ks and PDPK1 are negative regulators of CMA. Inhibition of class I PI3Ks with three structurally distinct, yet highly specific small molecules, is sufficient to activate CMA in cultured NIH3T3, AML12, and mIMCD3 cells, in the presence of serum. Similarly, inhibition of PDPK1 with three structurally distinct and specific small molecules is sufficient to activate CMA in the same cultured cell models (Endicott et al., 2020). Young adult mice treated with either buparlisib or pictilisib (class I PI3K inhibitors) showed increased uptake of endogenous CMA substrates into lysosomes, without changing macroautophagy (Endicott et al., 2020). Buparlisib and pictilisib have not been evaluated by lifespan studies in mice, but a similar compound, Alpelisib, has been used as a longevity intervention.

Class I PI3Ks include four catalytic subunits, PIK3CA (p110α), PIK3CB (p110β), PIK3CD (p110δ), and PIK3CG (p110γ), each with distinct expression profiles and interaction partners (Fruman et al., 2017). PIK3CA and PIK3CB are expressed ubiquitously and can associate with IRS1 to mediate INS and IGF1 pathway signaling (Fruman et al., 2017). However, PIK3CA is thought to play a more predominant role than PIK3CB in INS pathway activation (Fruman et al., 2017). PIK3CD and PIK3CG are expressed primarily in blood and immune cells, and their inhibition causes immunosuppression, an effect that does not arise from inhibiting PIK3CA and PIK3CB (Zhang et al., 2019). Thus, PIK3CA is likely to be the best target, among PI3K catalytic subunits, for diminishing INS and IGF1 signaling.

Alpelisib is an orally bioavailable inhibitor of PIK3CA, with high specificity over the other class I PI3K catalytic subunits (Furet et al., 2013; Hedges et al., 2021). Both sexes of C57BL/6J mice administered Alpelisib at a dose of 300ppm in food from 12 months of age live 10% longer than animals on a control diet (Hedges et al., 2023). The effects of alpelisib on healthspan are complex. Compared to mice on a control diet, alpelisib treated mice have side effects of hyperglycemia and hyperinsulinemia and an elevated risk of having hepatitis-like symptoms at time of necropsy (Hedges et al., 2021; Hedges et al., 2023). At 24 months of age, alpelisib treated mice of both sexes have higher grip strength, longer rotarod runs, and normal treadmill endurance, despite having reduced bone marrow density (Hedges et al., 2023). Future work will be needed to evaluate CMA in Alpelisib treated mice.

### 3.2 Constitutive activation of CMA in long-lived PTEN overexpressing mice

The PI3K/AKT/MTOR pathway is negatively regulated by a non-redundant lipid phosphatase, PTEN (Maehama and Dixon, 1999). PTEN converts PI(3,4,5)P_3_ to PI(4,5)P_2_, preventing further activation of AKT (Maehama and Dixon, 1998; Myers et al., 1998). The protein abundance of PTEN affects the rate of PI(3,4,5)P_3_ conversion to PI(4,5)P_2_, in turn regulating the duration of AKT activation (Mukherjee et al., 2021). Global overexpression (OE) of PTEN is sufficient to extend lifespan of both male and female mice (Ortega-Molina et al., 2012). PTEN OE mice show other signs of slowed aging and increased healthspan, such as improved brown adipose function, reduced DNA damage, and improved tightrope performance in 18–24 month-old animals (Ortega-Molina et al., 2012). PTEN OE reduces the incidence of spontaneous cancer (Ortega-Molina et al., 2012) and confers resistance to carcinogens (Garcia-Cao et al., 2012).

PTEN OE mice have increased hepatic CMA, with normal levels of macroautophagy (Zhang et al., 2023a). In cultured AML12 cells (hepatocytes), PTEN OE activates CMA in a manner that is dependent upon its lipid phosphatase activity, and which is blocked by a constitutively active AKT mutant (Zhang et al., 2023a). This suggests that PTEN activates CMA through canonical signaling mechanisms (Figures 5A, B). PTEN OE mice also have reduced liver levels of enzymes required for the generation of cytoplasmic acetyl-coA (ACLY and ACSS2) and *de novo* fatty acid synthesis (ACACA and FASN) (Zhang et al., 2023a). All four of these enzymes are CMA targets, whose protein abundance is tightly controlled by CMA (Endicott et al., 2022). This reduction in lipogenic enzymes might explain why PTEN OE mice do not become obese or develop fatty liver disease when fed a high-fat diet (Ortega-Molina et al., 2012).

PTEN deficiency is implicated in the pathogenesis of autism, diabetes, and most types of cancer (Keniry and Parsons, 2008). When *Pten* is depleted in AML12 cells by siRNA, CMA is strongly inhibited, which can be reversed by treatment with PI3K inhibitors (Zhang et al., 2023a). Mice with a liver-specific *pten* deletion and mice with a liver-specific deletion of CMA receptor *lamp2a* show remarkably similar phenotypes, including spontaneous development of fatty liver disease, reductions in peripheral adiposity, and a shift in liver energy production favoring glycolysis over oxidative phosphorylation (Stiles et al., 2004; Schneider et al., 2014). These findings suggest the testable hypothesis that some of the phenotypes arising in pten-deficient mice might arise from a decrease in CMA.

### 3.3 Constitutive activation of CMA in long-lived endocrine mutant mice

Mice with genetic ablation of GH production, such as the hypopituitary *pou1f1* mutant (Snell dwarf) or ablation of GHR, such as the *ghr* KO mouse, are dwarves with reduced circulating INS and IGF1 levels, low rates of lethal cancer, altered carbohydrate metabolism, increased INS sensitivity, and significant lifespan extension (Flurkey et al., 2001; Coschigano et al., 2003; Anisimov and Bartke, 2013). Consistent with the decreases in circulating INS and IGF1, both Snell dwarf and *ghr* KO mice have reduced phosphorylation of AKT (on S473) in the liver, compared to their normal siblings, when fed (Dominick et al., 2015).

*ghr* KO mice have normal (or slightly increased, depending on the method of measurement) macroautophagy in the liver, compared to normal siblings, when fed *ad libitum*. *ghr* KO mice have clearly enhanced CMA (Endicott et al., 2021). Snell dwarf mice have decreased hepatic macroautophagy, and increased CMA, when fed *ad libitum* (Endicott et al., 2021). These data were the first to suggest that CMA is controlled by endocrine signals, and that those signals are distinct from the endocrine signals that regulate macroautophagy.

Because both Snell dwarf mice and *ghr* KO mice have reduced signaling through the GH pathway, it was initially hypothesized the GH was a negative regulator of CMA. However, mice with a liver-specific deletion of GHR, which have normal lifespans (Dominick et al., 2015), do not have increased CMA in the liver (Endicott et al., 2021). This suggests that enhanced CMA in Snell and *ghr* KO mice is not a direct effect of reduced GH signaling, but rather, is an effect of secondary endocrine changes that occur downstream of a loss of GH (as in Snell dwarf) or GHR (as in *ghr* KO). This led to the hypothesis that diminished INS and IGF1, rather than GH, is responsible for enhanced CMA in Snell and *ghr* KO mice, leading to subsequent studies of CMA in PTEN OE mice, and mice treated with inhibitors of class I PI3Ks.

### 3.4 CMA is enhanced in mice and rats subjected to calorie restriction

Calorie restriction (without malnutrition) is the most reliable way to extend metazoan lifespan across phyla (Anderson and Weindruch, 2010). Male Fisher-344 rats were fed *ad libitum* or subjected to 40% calorie restriction, starting at age 16 weeks. Liver lysosomes were isolated at ages 4, 12, and 22 months, and CMA activity was measured *in vitro* (Jafari et al., 2024). As previously reported, the proteolysis of CMA substrate proteins was decreased in old rats, compared to young (Cuervo and Dice, 2000; Jafari et al., 2024). However, lysosomes isolated from rats that had been calorie restricted had higher CMA activity than those from *ad libitum* fed mice at ages 12 or 22 months (Jafari et al., 2024). This suggests that calorie restriction at least partially protects against the age-related decline in CMA in male Fisher-344 rats.

NIH3T3 cells expressing the fluorescent CMA reporter (KFERQ-Dendra2) were cultured in media supplemented with serum from rats that were fed *ad libitum* or calorie restricted. The cells grown in serum for calorie restricted rats had higher CMA activity than those grown in serum from *ad libitum* fed rats (Jafari et al., 2024). This suggests that changes in circulating endocrine factors in response to calorie restriction, rather than cell-intrinsic changes, might be responsible for the enhanced CMA in calorie restricted rats (Jafari et al., 2024). Because calorie restriction reduces circulating INS and IGF1 (Al-Regaiey et al., 2005), it is reasonable to hypothesize that diminished signaling through the INS/IGF1 pathway is responsible for enhanced CMA in calorie restricted mice.

## 4 CMA target proteins regulate aging

### 4.1 MYC

MYC (specifically c-MYC) is a vital regulator of stem cell pluripotency (Takahashi et al., 2007) and a positive regulator of anabolism and cell growth (Stine et al., 2015; Duffy et al., 2021). MYC primarily functions as a transcription factor, and its expression is tightly controlled in healthy cells. MYC expression is elevated in as many as seventy percent of cancers, and it is one of the most commonly studied targets for cancer therapy (Stine et al., 2015; Duffy et al., 2021).

C57BL/6 mice that are hemizygous for the MYC gene have reduced adiposity, delays in age-related loss of bone density, and fewer age-related transcriptional changes in the liver, white adipose, and skeletal muscle, compared to their normal siblings (Hofmann et al., 2015). MYC hemizygous mice have increased median lifespans (21% gain for females, and 11% for males). Both sexes have normal reproductive outputs (Hofmann et al., 2015). MYC hemizygous mice also show reduced circulating levels of IGF1 (Hofmann et al., 2015).

MYC is indirectly controlled by CMA. CMA targets and degrades CIP2A (cellular inhibitor of PP2A), which in turn enhances PP2A activity (Gomes et al., 2017). PP2A dephosphorylates MYC, enhancing its degradation by the proteasome (Gomes et al., 2017). Consistent with these findings, Snell dwarf mice, which have enhanced CMA, have decreased protein levels of CIP2A and MYC in liver, kidney, and skeletal muscle, with no changes in the abundances of the mRNAs that encode these proteins (Endicott et al., 2021). PTEN OE mice (which also have elevated CMA) have reduced MYC protein levels in the liver, lung, spleen, white adipose, and brown adipose (mRNA levels not reported) (Garcia-Cao et al., 2012). These data suggest the intriguing hypothesis that a reduction in MYC, as a consequence of enhanced CMA, could contribute to the exceptional longevity of Snell dwarf and PTEN OE mice.

### 4.2 NLRP3

NLRP3 is a sensor of cellular damage that is triggered by a wide variety of cellular stressors, including infection (Torre-Minguela et al., 2017). After detecting a cellular stress, NLRP3 oligomerizes into the NLRP3 inflammasome, a signaling platform that triggers downstream inflammatory signaling events such as activation of caspase 1 and production of IL-1β (Torre-Minguela et al., 2017). Increased activation of NLRP3 with age is thought to contribute to an age-related increase in sterile inflammation, commonly referred to as “inflammaging” (Sebastian-Valverde and Pasinetti, 2020).

C57BL/6 mice with a homozygous knockout of NLRP3 gain a 34% increase in median lifespan, when sexes are pooled (Marín-Aguilar et al., 2020). Functional analyses, performed only in male mice, showed that NLRP3 deficiency prevents the age associated increase in left ventricular hypertrophy and fibrosis (Marín-Aguilar et al., 2020). A separate study evaluating the effects of NLRP3 on female reproductive longevity obtained similar results–female C57BL/6J mice with a homozygous knockout of NLRP3 had a 37% increase in median lifespan (Navarro-Pando et al., 2021). NLRP3 expression increases as a function of age in ovaries of wildtype female C57BL/6J mice, which co-occurs with an age-related increase in protein levels of caspase 1 and IL-1β (Navarro-Pando et al., 2021). NLRP3 KO female mice show delays in age-related changes in circulating reproductive hormones and have more follicles in their ovaries at 12 months of age, compared to wildtype controls. At 4 months of age, almost all wildtype and NLRP3 KO females are able to become pregnant. By 12 months of age, less than half of wildtype females can become pregnant, but NLRP3 KO females have pregnancy rates similar to 4-month-old animals (Navarro-Pando et al., 2021). At ages 4 and 12 months, NLRP3 KO females have significantly larger litter sizes than WT controls (Navarro-Pando et al., 2021).

Several recent papers have identified NLRP3 as a CMA target, and have shown that CMA downregulates inflammation through the selective degradation of NLRP3 (Qiao et al., 2021; Wang et al., 2023; Wu et al., 2023). These findings suggest the intriguing possibility that future small molecule interventions that selectively activate CMA could promote female reproductive longevity by degrading NLRP3.

To our knowledge, there have not been studies characterizing changes to the NLRP3 inflammasome in mice with elevated CMA. However, these studies are well-justified given the clear evidence that NLRP3 is both a target of CMA degradation and a regulator of mouse longevity and reproductive lifespan.

### 4.3 ACLY and ACSS2

Acetyl-coA is most well-known as the carbon source for the tricarboxylic acid cycle in mitochondria. However, it also plays important roles in the cytoplasm and nucleus, as an essential reactant in protein acetylation (including histone acetylation) and *de novo* lipogenesis (Pietrocola et al., 2015). Acetly-coA cannot be exchanged between the mitochondrial and nucleocytosolic compartments. Thus, acetyl-coA must be produced outside of the mitochondria by one of two enzymes, ACLY and ACSS2. ACLY consumes citrate, yielding acetyl-coA and oxaloacetate. ASSC2 produces acetyl-coA from acetate (Pietrocola et al., 2015).

Male fruit flies hemizygous for the *Drosophila* ortholog of ACLY show a 32% increase in median lifespan and a decrease in several acetyl-lysine histone marks (Peleg et al., 2016). It is hypothesized that the reduction in ACLY might extend *Drosophila* lifespan by preventing an age-related increase in transcriptional noise (Peleg et al., 2016). Both male and female flies treated with RNAi targeting the *Drosophila* ortholog of ACSS2 have a significant extension of median lifespan (Eisenberg et al., 2014).

It is unclear how rodent lifespan will be affected by genetic alterations that globally reduce in ACLY and ACSS2. ACLY and ACSS2 play complex tissue- and cell-type specific roles in metabolism and gene regulation in mammals (Bradshaw, 2021). Thus, tissue-specific knockouts of these genes might be required to fully understand their roles in regulating lifespan. However, the naturally occurring ACLY inhibitor, hydroxycitrate, has properties of a calorie-restriction mimetic, and this compound can reduce adiposity and insulin resistance in obese Zucker rats (Madeo et al., 2019). Similarly, inhibition of ACLY with bempedoic acid reduces lipid droplet accumulation and fibrosis in mouse models of non-alcoholic fatty liver disease (C57BL/6 mice on high-fat, high-fructose diet) (Morrow et al., 2022). Male C57BL/6 mice treated with a high dose (7,500 ppm) of hydroxycitrate in chow showed a significant reduction in early mortality, but no significant change in median lifespan (Espadas et al., 2024).

The protein levels of ACLY and ACSS2 are highly sensitive to CMA in cultured AML12 (hepatocytes) and NIH3T3 (mouse embryonic fibroblasts) cells, and this is independent from changes in mRNA levels (Endicott et al., 2022). Both ACLY and ACSS2 show increased targeting to the liver lysosomes in *ghr* KO mice and PTEN OE mice (Endicott et al., 2022; Zhang et al., 2023a). This increase in lysosomal targeting is mirrored by a decrease in total liver levels of these two proteins in *ghr* KO and PTEN OE mice (Endicott et al., 2022; Zhang et al., 2023a). Similarly, Snell dwarf mice show reduced liver levels of these two proteins (Endicott et al., 2022).

PTEN OE mice do not develop fatty liver disease, obesity, and insulin resistance when fed a high fat diet, even though their normal siblings develop all of these phenotypes (Ortega-Molina et al., 2012). Whether or not the CMA-mediated reduction in ACLY and ACSS2 confers PTEN OE mice protection from the negative effects of a high-fat diet on liver health has not yet been evaluated, and will need to be addressed by future work. Additional future studies will be needed to assess if ACLY and ACSS2 are similarly altered by other CMA activating interventions, and how these proteins change in tissues beyond the liver.

## 5 Changes in CMA with age

### 5.1 Important considerations for designing experiments to study aging in rodents

There are several important design considerations that should be applied when planning experiments to study age-associated biological changes in rodents. These considerations are presented in great detail in commentaries by Miller and colleagues (Miller et al., 1999; Miller and Nadon, 2000). There are two considerations especially relevant to studies of age-related changes in CMA, (1) to use appropriate genetic models, or at the very least, to avoid generalizing findings from inbred strains to the species as a whole and (2) to avoid using mice that are much older than the median lifespan of their genetic background.

Mouse strains bred to homozygosity at every gene are commonly used for biomedical research. However recent genetic analyses comparing related, but genetically distinct, stocks of inbred mice have decisively demonstrated that results obtained from any single isogenic rodent stock cannot safely be generalized to other isogenic or inbred stocks, or to mice in general, because results observed in any one such stock are frequently not replicated in other stocks, even when tested in the same laboratory at the same time (Miller, 2016; Sittig et al., 2016; Neuner et al., 2019; Sleiman et al., 2022). Individuals of inbred mouse strains frequently develop the same strain-specific lethal cancer (Tam and Cheung, 2020). These cancers can be major confounding factors when attempting to interpret observed changes with age. For example, over one-third of C57BL/6J mice die of lymphoma, and half of C57BL/6J mice have lymphoma at time of their death, even if it was not the proximal cause of death (Brayton et al., 2012). Thus, a researcher evaluating age-related changes in lymphocytes (B cells, T cells, etc.) in C57BL/6J mice would very likely notice significant differences between young and old animals. However, it would not be responsible to claim that any observed differences in lymphocytes arise from aging, *per se*, when these differences could also be explained by the neoplastic transformation of lymphocytes into cancer.

Mice (or rats) that are older than the median lifespan of their genetic stock (“very old”) should be avoided (Miller and Nadon, 2000). It is not possible to say if biological differences observed between very old mice and young adult controls are the consequences of normal aging, or simply manifestations of terminal illness present only at the final stages of life. Moreover, when using very old animals of a genetically diverse stocks, then selection bias must be considered, i.e., differences observed between very old animals and young controls might not be results of aging, but instead be factors that promote longevity. For example, in the genetically heterogeneous UM-HET3 mice stock, some quantitative trait loci on chromosome 12 are uniquely found in mice that live to very old age (Sleiman et al., 2022), but these genes do not change as a function of age. In addition to avoiding very old animals, studies of age-related biological changes can also be improved by including a group of middle-aged animals, in addition to the young controls (Miller and Nadon, 2000).

### 5.2 Effects of age on CMA and CMA-related proteins

Several recent review papers have stated that CMA decreases with age in most cell types and tissues of mice and rats (Cuervo and Wong, 2014; Kaushik and Cuervo, 2015; Tasset and Cuervo, 2016; Xilouri and Stefanis, 2016; Kaushik et al., 2021). According to this paradigm, the main factor responsible for the age-related decline in CMA is a reduction in LAMP2A, which is required for the uptake of CMA target proteins into the lysosome. The abundance of the *Lamp2a* transcript is not changed with age (Kiffin et al., 2007; Valdor et al., 2014). Rather, the stability of the LAMP2A protein at the lysosome is reduced, which is attributed to an age-related change in lysosomal membrane lipid composition (Kiffin et al., 2007; Rodriguez-Navarro and Cuervo, 2012; Rodriguez-Navarro et al., 2012). Here, we describe the results from every paper (that we are aware of) that measures CMA, or CMA related proteins, in rodents at more than one age group. Results are summarized in Table 1.

**TABLE 1:** Age-associated changes in CMA and CMA-related proteins. A summary of key results from papers that have compared CMA or CMA-related proteins in mice or rats of multiple age groups.

PMID	Genetic background		Sex	Young	Middle	Old	Cell/Tissue	Assay	Ages compared	Effect of age	Median lifespan
10806201	*R.n.*	Fisher-344	M	3	—	22	Liver lysosomes	Exogenous Substrate Uptake	22 v 3	Reduced	23
							Liver lysosomes	WB: LAMP2A	22 v 3	Reduced	
							Whole liver	WB: LAMP2A	22 v 3	Unchanged	
18690243	*M.m.*	FVB	M	6	—	22	Liver lysosomes	Exogenous Substrate Uptake	22 v 6	Reduced	19
							Liver lysosomes	WB: LAMP2A	22 v 6	Reduced	
							Whole liver	WB: LAMP2A	22 v 6	Reduced	
23521856	*M.m.*	C57BL/6J	—	3		22	Retina	Turnover of ^3^H labeled proteins	22 v 3	Increased	25–28
							Retina	WB: LAMP2A	22 v 3	Increased	
25263126	*M.m.*	C57BL/6	—	4		22	CD4+ T cells	WB: LAMP2A	22 v 4	Reduced	25–28
28238968	*M.m.*	C57BL/6J	M	3.5–7		24–29	Skeletal muscle	WB: LAMP2A	24 v 3.5	Reduced	25–28
							Cardiac muscle	WB: LAMP2A	24 v 3.5	Increased	
33442062	*M.m.*	C57BL/6J	M	4	12	30	HSCs	KFERQ-Dendra2 Reporter	12 v 4	Unchanged	25–28
							HSCs	KFERQ-Dendra2 Reporter	30 v 4	Reduced	
							HSCs	IF: LAMP2A	30 v 4	Reduced	
							GMPs	KFERQ-Dendra2 Reporter	30 v 4	Unchanged	
37315291	*M.m.*	UM HET3	M&F	4	14	24	Whole liver	WB: LAMP2A	all	Increased	M: 25–28
							Whole kidney	WB: LAMP2A	all	Unchanged	F: 28–29
							Whole brain	WB: LAMP2A	24 v 4	Unchanged	
							Liver lysosomes	WB: LAMP2A	24 v 4	Unchanged	
							Liver lysosomes	Exogenous Substrate Uptake	24 v 4	Unchanged	
	*M.m.*	C57BL/6J	M	2	8	24	Whole liver	WB: LAMP2A	all	Increased	25–28

An age-related decline in CMA was first reported in 2000, when isolated liver lysosomes from 20-h fasted 22-month-old male Fisher-344 rats were found to have reduced uptake of CMA substrates compared to lysosomes from 3-month-old controls (Cuervo and Dice, 2000). The lysosomes from old animals were reported to show increased levels of lumenal enzymes, such as cathepsins and hexosaminidase, while showing decreased levels of LAMP2A (Cuervo and Dice, 2000). The decrease in lysosomal LAMP2A in 22-month-old animals, relative to 3-month-old animals, was not due to a change in total liver levels of LAMP2A, which remained the same between the age groups (Cuervo and Dice, 2000). The median lifespan of *ad libitum* fed male Fisher 344 rats is 714 days (23 months) (Yu et al., 1982).

Male Fisher-344 rats at ages 4, 12, and 22 months do not show differences in the mRNA levels of *Lamp2a* (Kiffin et al., 2007). However, there are age-related changes in LAMP2A dynamics at the lysosomal membrane. In 4-month-old rats, a substantial portion of lysosomal LAMP2A resides in detergent-resistant lipid rafts. However, in 22-month-old rats, the amount of LAMP2A in detergent resistant lipid rafts is dramatically reduced. This coincides with an age-related shift of LAMP2A from the lysosomal membrane to the lysosomal matrix (Kiffin et al., 2007). The change in the distribution of LAMP2A between lysosomal compartments, as a function of age, has been interpreted to mean that the stability of LAMP2A at the lysosomal membrane is compromised in old rats, leading to its enhanced degradation. This in turn explains a reduction in lysosomal levels of LAMP2A with age, despite unchanged mRNA levels (Kiffin et al., 2007).

Both whole liver lysates and liver lysosomes from male FVB mice at 22 months of age have sharp reductions in LAMP2A protein levels, compared to samples derived from 6-month-old controls. Lysosomes from 22-month-old animals also showed significantly less uptake of GAPDH than lysosomes from 6-month-old animals (Zhang and Cuervo, 2008). In the laboratory reporting these findings, the median lifespan of male FVB mice is 19 months (Zhang and Cuervo, 2008). A prior study evaluating spontaneous tumor development in FVB mice found that 13% of 14-month-old males and 55% of 24-month-old males had tumors (Mahler et al., 1996). 31% of 24-month-old males had abnormalities noted in the liver, with 21% showing tumors and an additional 10% showing “foci of cellular alteration” observed by H&E staining (Mahler et al., 1996). Thus, caution should be applied when interpreting whether the observed decrease in CMA results entirely from aging, from cancer, or a combination of both.

Evidence for age-related decline in CMA has also been reported in hematopoietic stem cells (HSCs). HSCs isolated from C57BL/6 male mice expressing globally expressing the KFERQ-Dendra2 reporter were assessed for the presence of fluorescent puncta, at ages 4, 12, and 30 months. There was no difference in CMA puncta in HSCs between 4- and 12-month-old animals, but 30-month-old animals showed a significant reduction in puncta. Granulocyte-monocyte progenitor cells did not show any difference in CMA puncta between 4- and 30-month-old animals (Dong et al., 2021). The median lifespan of wild type male C57BL/6J mice, fed *ad libitum*, varies slightly between papers, but is typically 25–28 months (Pyo et al., 2013; Fernández et al., 2018; Hedges et al., 2023).

An age-related decline in CMA has been reported in T cells. CD4^+^ T cells were isolated from 4-month-old C57BL/6J mice of unspecified sexes, and the cells were stimulated for 24 h with anti-CD3 and anti-CD28 (Valdor et al., 2014). This treatment caused an increase in LAMP2A protein levels in CD4^+^ T cells from 4-month-old animals. However, this induction failed to occur in CD4^+^ T cells isolated from 22-month-old animals (Valdor et al., 2014). Over one-third of C57BL/6J mice die of lymphoma, and half of C57BL/6J mice have lymphoma at time of their death (Brayton et al., 2012). Thus, observed changes in CMA in T cells from very old C57BL/6 mice could be an effect of aging, lymphoma, or both.

Even though the prevailing perspective is that CMA declines with age (Cuervo and Wong, 2014; Kaushik and Cuervo, 2015; Tasset and Cuervo, 2016; Xilouri and Stefanis, 2016; Kaushik et al., 2021), there are some papers reporting contrary evidence. A 2013 study found that the retinas of 22-month-old and 12-month-old C57BL/6J mice of unspecified sexes showed reduced LC3 flux (indicating reduced macroautophagy) compared to retinas from 3-month-old animals (Rodríguez-Muela et al., 2013). Despite the reduction in LC3 flux, metabolic labeling experiments found that there is increased turnover of long-lived proteins in retinas of 22-month-old animals compared to 3-month-old animals (Rodríguez-Muela et al., 2013). This increased protein turnover might be attributable to CMA, because retinas from 12-month-old and 22-month-old animals have higher expression of LAMP2A than retinas from 3-month-old animals (Rodríguez-Muela et al., 2013).

Male C57BL/6J mice have reduced protein levels of LAMP2A and HSPA8 in skeletal muscle in old age (24–29 months), compared to young mice (3.5–7 months) (Zhou et al., 2017). However, the same mice have increased expression of LAMP2A in the heart in old age, with no change in HSPA8 (Zhou et al., 2017). This study has a few limitations; the “old” animals were beyond the median lifespan of the stock, and changes in the abundance of LAMP2A and HSPA8 are not sufficient to prove changes in CMA activity. However, this study indicates that age-related changes in LAMP2A protein levels are tissue-specific.

We recently evaluated CMA in both female and male UM-HET3 mice at 4, 14, and 24 months of age. UM-HET3 mice are the four-way cross used by the NIA Interventions Testing Program. There were no changes in the protein levels of LAMP2A or HSPA8 in the liver, kidney, or brain of UM-HET3 mice. However, female UM-HET3 mice had significantly higher LAMP2A levels in the brain than males (Zhang et al., 2023b). There were no differences in the lysosomal levels of LAMP2A or HSPA8 between 4- and 24-month-old animals of either sex, in either the fed or fasted nutritional states (Zhang et al., 2023b). Moreover, we found no age-related difference the ability of liver lysosomes to internalize CMA substrate MAPT (Zhang et al., 2023b). The median lifespan for UM-HET3 mice varies slightly between cohorts and testing sites, but is typically 25–28 months for males, and 28–29 months for females (Miller et al., 2011; Harrison et al., 2021; Harrison et al., 2024). Thus, all age groups used in our study were under the median lifespan.

It is clear that age-related changes in LAMP2A and/or CMA activity are context-dependent. Contexts that seem to affect age-related changes in CMA and LAMP2A include (1) the genetic background of the animals, (2) the tissue or cell type under consideration, and (3) the ages at which the animals are evaluated. Future research should address the contribution of biological sex to the effects of age on CMA.

## 6 Loss of CMA causes disease

A loss of function in CMA has been implicated in the pathogenesis of atherosclerosis (Qiao et al., 2021), fatty liver disease (Schneider et al., 2014), diabetes-associated kidney hypertrophy (Sooparb et al., 2004), cancer (Schneider et al., 2015; Xia et al., 2015; Gomes et al., 2017), and neurodegeneration (Xilouri et al., 2016; Bourdenx et al., 2021). For readers interested in the roles of CMA in cell, tissue, and organ biology, beyond the scope of the aging field, there are several excellent papers available. CMA prevents renal tube hypertrophy (Franch, 2014), facilitates CD4^+^ T cell activation (Valdor et al., 2014), modulates DNA-damage responses (Park et al., 2015a), is involved in the response to traumatic brain injury (Park et al., 2015b), and combats Huntington’s disease, and may be responsible for reduced cancer in Huntington’s patients (Hasholt, 2023).

A loss of CMA might be involved in the pathogenesis of Danon Disease, which is caused by mutations that deplete all *Lamp2* isoforms, or that specifically deplete *Lamp2b* (Nishino et al., 2000). Danon disease is phenotypically characterized by hypertrophic cardiomyopathy, skeletal myopathy, and intellectual disability (Endo et al., 2015), with a median lifespan of 19 years for men, and 35 years for women (Boucek et al., 2011). The possible roles for LAMP2A and LAMP2C in Danon disease should not be overlooked. Patients with mutations to exon 9B of *Lamp2* (ablating only *Lamp2b*) have less severe phenotypes and longer survival times than patients with mutations affecting all *Lamp2* variants (Hong et al., 2012). Thus, it is likely to be the case that the phenotypes arising from the most severe forms of Danon disease (when a shared exon is mutated) result from the combined effects of losing LAMP2A, LAMP2B, and LAMP2C proteins.

A loss of CMA can contribute to the pathogenesis of age-related disease via the dysregulated abundance of specific proteins, through impairment of proteostasis, or a combination of the two. CMA plays an important role in cellular stress resistance. NIH3T3 cells depleted of LAMP2A are sensitive to a variety of stressors–they have reduced viability, compared to WT cells, when exposed to hydrogen peroxide, paraquat, ultraviolet light, and a 42°C heat shock. There are multiple possible explanations of this result. CMA might degrade damaged and oxidized proteins, providing protection against cellular stressors. Alternatively, after a stressor occurs, CMA might degrade proteins that are not beneficial to cell viability during a stress, such as those that promote cell growth, division, or apoptosis. It is also worth noting that CMA deficient cells are metabolically distinct from normal cells (Schneider et al., 2014; Zhang et al., 2023a), which might make them more vulnerable to some categories of stress.

There is some support for the idea that CMA degrades damaged and oxidized proteins. Male C57BL/6 mice with a liver-specific knockout of *Lamp2a* have increased abundances of oxidized proteins and ubiquitinated proteins in the liver, and they have higher levels of reactive oxygen species in the liver (Schneider et al., 2015). This suggests that blocking CMA severely disrupts protein quality control. Similarly, in male C57BL/6 mice lacking *Lamp2a* in excitatory neurons, there is increased protein oxidation and ubiquitination in the hippocampus (Bourdenx et al., 2021). CMA is also known to play a role in protein quality control. For example, CMA degrades newly synthesized H3 and H4 in HeLa cells. H3, which contains a mutation that prevents it from stably interacting with H4, is preferentially degraded by CMA, suggesting that CMA might play an important role in quality control of newly synthesized histone proteins (Hormazabal et al., 2022).

### 6.1 Cancer

Cancer has historically been viewed as a genetic disease. However, recent advances have shown that there are metabolic, epigenetic, and endocrine events that contribute to neoplastic transformation, cancer evolution, and therapy resistance (Marine et al., 2020; Shlyakhtina et al., 2021). CMA is essential for maintaining a healthy metabolism (Schneider et al., 2014), and CMA is essential for regulating the protein levels of ACLY and ACSS2 (Endicott et al., 2022), which provide acetyl-coA for histone acetylation (and which can be oncogenic if overexpressed) (Hao et al., 2022). Male C57BL/6 mice with a liver-specific deletion of LAMP2A spontaneously develop liver tumors with age. Twenty-seven percent have liver tumors by 24 months of age, with no liver tumors found in controls (Schneider et al., 2015). Thus, loss of CMA contributes to cancer development. Consistent with the idea that CMA might have a tumor-suppressor function, three types of long-lived mice with elevated CMA (Snell dwarf, ghr KO, and PTEN overexpressor) all have reduced frequencies of spontaneous cancers (Ortega-Molina et al., 2012; Anisimov and Bartke, 2013).

CMA may negatively regulate neoplastic transformation by degrading proteins with oncogenic potential, by promoting a tumor-suppressive metabolic state, or a combination of the two. However, we will focus on proteins degraded by CMA that have oncogenic potential. These proteins include CIP2A (Gomes et al., 2017), MDM2 (Lu et al., 2010), mutant TP53 (Vakifahmetoglu-Norberg et al., 2013), TPT1 (also called TCTP) (Bonhoure et al., 2017), and HK2 (Xia et al., 2015).

CIP2A is an endogenous inhibitor of protein phosphatase 2A (PP2A). PP2A is a tumor suppressor that negatively regulates signaling through several pro-growth pathways including the PI3K/AKT/MTOR, MEK/ERK, and WNT/β-catenin (Soofiyani et al., 2017). PP2A also dephosphorylates MYC, which makes it susceptible to ubiquitination and proteasomal degradation (Soofiyani et al., 2017). PP2A is frequently inactivated in cancers. However, that inactivation is rarely caused by mutations in the genes that encode the protein subunits of PP2A. Rather, PP2A is inactivated in cancer cells by increased expression of PP2A inhibitor proteins, like CIP2A (Kauko and Westermarck, 2018). The degradation of CIP2A by CMA promotes PP2A activity and downregulates MYC protein levels (Gomes et al., 2017).

MDM2 is an E3 ubiquitin ligase that negatively regulates the protein levels of TP53 (p53) (Chinnam et al., 2022). MDM2 expression is positively regulated by TP53. Thus, MDM2 is part of a negative feedback loop that keeps TP53 protein levels low (Koo et al., 2022). CMA degrades MDM2 (Lu et al., 2010). The interaction of MDM2 is enhanced by the presence of TPT1, which prevents MDM2 autoubiquitination, and increases the efficiency of TP53 ubiquitination by MDM2 (Amson et al., 2012). Like MDM2, TPT1 protein levels are negatively regulated by CMA degradation. TP53 loss of function mutations have been identified in many types of cancer. However, some TP53 mutations lead the to the expression of full-length protein with dominant-negative effects on TP53 function. CMA selectively degrades some of these mutant forms of TP53, allowing the remaining wild-type TP53 to exert its tumor suppressor activity (Vakifahmetoglu-Norberg et al., 2013). Thus, it can be hypothesized that CMA promotes TP53 tumor suppressor function by degrading MDM2, TPT1, and at least some forms of dominant-negative TP53.

Finally, CMA degrades HK2 (Hexokinase II), which is involved in the initial stages of glycolysis (Xia et al., 2015). Non-acute myeloid leukemia cells become more sensitive to inhibitors of the class III receptor tyrosine kinase FLT3 when macroautophagy is inhibited. The mechanism of action is that FLT3 inhibition activates CMA, and that activation is boosted by inhibition of macroautophagy. CMA activation in turn reduces HK2 levels, causing a metabolic catastrophe for the cancer cells (Xia et al., 2015).

Despite the evidence that CMA might reduce the risk of neoplastic transformation, it should be noted that CMA has a complex relationship with cancer. High levels of CMA in healthy cells/tissues may reduce the frequency of neoplastic transformation by degrading proteins that have oncogenic potential. However, after a transformation event, CMA may promote cancer by helping some kinds of cancer cells to sustain their energy requirements (Hubert et al., 2022).

### 6.2 Neurodegeneration

The relationship between CMA and neurodegeneration is complex. In healthy cells, CMA degrades proteins implicated in the pathophysiology of Alzheimer’s, Parkinson’s, and Huntington’s diseases. However, CMA is inhibited by mutant forms of MAPT, LRRK2, and SNCA. This loss of CMA might promote neurodegeneration by causing a broad dysregulation of protein degradation and proteotoxic stress (Kaushik and Cuervo, 2018).

Mice with LAMP2A specifically deleted in excitatory neurons (from CRE expressed under a CAMK2A promoter) have shortened lifespans, and by 6 months of age, they show declines in neuronal proteostasis, declines in spatial memory (Y-maze test), and reduced nesting behavior (Bourdenx et al., 2021). Moreover, proteins that are prone to aggregation (such as SNCA, MAPT, UCHL1, and PARK7) were more likely to be found in the insoluble fraction taken from brains of mice with excitatory neuron LAMP2A deletion (Bourdenx et al., 2021). These data collectively suggest that neurodegeneration manifests by young adulthood for mice lacking LAMP2A in excitatory neurons.

The first neurodegenerative disorder to be associated with CMA was Parkinson’s disease. CMA degrades wildtype human SNCA, but SNCA with pathogenic mutations A53T or A30P binds to LAMP2A and prevents the degradation of both SNCA and other CMA substrates (Cuervo et al., 2004). Other Parkinson’s disease-associated proteins that are degraded by CMA include PARK7 (Wang et al., 2016), LRRK2 (Orenstein et al., 2013), and UCHL1 (Kabuta et al., 2008). CMA also degrades proteins associated with Alzheimer’s disease and frontotemporal dementia, such as MAPT (Tau) (Wang et al., 2009), TARDBP (Huang et al., 2014), and APP (Park et al., 2016).

When mice with a systemic knockout of LAMP2A are crossed with the triple transgenic Alzheimer’s model (expressing pathogenic variants of MAPT, APP, and PSEN2), the hybrid animals show a greatly exacerbated neurodegeneration phenotype (including deposition of amyloid plaques), compared to the triple transgenic model alone (Bourdenx et al., 2021). Collectively, these findings point toward a hypothesis that activation of CMA could slow or delay the progression of neurodegenerative disease.

Small molecule antagonists of the retinoic acid receptor α (RARA) can increase CMA activity by stimulating the transcription of the *Lamp2a* mRNA (Anguiano et al., 2013; Gomez-Sintes et al., 2022). RARA antagonists, when injected into mice, can increase the association of the KFERQ-Dendra2 reporter with lysosomes in many tissues and cell types (Dong et al., 2020). When mice expressing a pathogenic mutant of human MAPT (PS19 mice), are treated with the RARA antagonist AR7 by daily oral administration, they show a normalization in the hyperactivity commonly observed in PS19 mice (Bourdenx et al., 2021). Moreover, PS19 mice treated with AR7 have significantly reduced accumulation of MAPT in the hippocampus, amygdala, and piriform cortex, compared to the untreated PS19 controls (Bourdenx et al., 2021). Similarly, triple transgenic Alzheimer’s model mice also showed delayed onsets of neurodegenerative phenotypes when administered AR7 (Bourdenx et al., 2021).

While the evidence that CMA activation can delay the progression of age-associated neurodegeneration is limited, it is also very compelling, and it certainly warrants additional studies. It is also clear that a loss of CMA in the brain is sufficient to cause spontaneous neurodegeneration, which is exacerbated in mice with pathogenic transgenes.

### 6.3 Fatty liver disease

Mice with a liver-specific *Pten* deletion [PTEN is a positive regulator of CMA (Zhang et al., 2023a)] and mice with a liver-specific deletion of CMA receptor LAMP2A show remarkably similar phenotypes, i.e., spontaneous development of fatty liver disease, reductions in peripheral adiposity, and a shift in liver energy production favoring glycolysis over oxidative phosphorylation (Stiles et al., 2004; Schneider et al., 2014). In both cases, the metabolic dysregulation of the liver eventually progresses to cancer (Stiles et al., 2004; Schneider et al., 2014). Mice overexpressing PTEN have enhanced CMA (Zhang et al., 2023a), and shift in metabolism to favor oxidative phosphorylation over glycolysis (Garcia-Cao et al., 2012). Moreover, mice overexpressing PTEN do not develop fatty liver disease or insulin resistance when fed a high-fat diet (Ortega-Molina et al., 2012), which is the stark opposite phenotype of LAMP2A- or PTEN-deficient mice.

While the mechanism linking CMA to fatty liver disease has not been completely resolved, experiments in cultured hepatocytes shed important clues. AML12 cells (normal mouse hepatocytes) have increased abundance of enzymes involved in *de novo* lipogenesis, when *Lamp2a* is depleted by siRNA (Endicott et al., 2022; Zhang et al., 2023a). Furthermore, overexpression of PTEN reduces the abundance of *de novo* lipogenesis enzymes and lipid droplet accumulation, which is blocked by knocking down *Lamp2a* (Zhang et al., 2023a). These results suggest the hypothesis that CMA downregulates lipid storage by degrading enzymes for *de novo* lipogenesis. Consistent with this hypothesis, PTEN overexpressing mice have reduced expression of *de novo* lipogenesis enzymes in the liver, but the same enzymes are increased in the livers of mice with liver-specific *Pten* deletion (Zhang et al., 2023a).

### 6.4 Other CMA-related phenotypes

CMA is an integral part of the feedback loop that regulates the abundance of the transcription factors that govern circadian rhythms. Male C57BL/6J mice with a global deletion of LAMP2A have perturbations in the protein levels of circadian transcription factors, such as BMAL and CLOCK, which are degraded by CMA, and which also regulate the transcription of CMA-related genes (Juste et al., 2021). Circadian rhythms become dysregulated with age, and, in some ways, the changes in circadian transcription factor expression in young (4-month-old) LAMP2A knockout mice resemble the changes observed in old (24-month-old) animals (Juste et al., 2021). Thus, it might be the case that there is an interplay between circadian dysregulation and an age-related decline in CMA, at least in the strain- and tissue-specific contexts where CMA declines with age.

## 7 Discussion

The two models explaining the relationship between CMA and aging both have several strengths and limitations that will need to be addressed by future research projects.

### 7.1 Discussion of the longevity model

Four kinds of long-lived mice have enhanced CMA, including Snell dwarf, *ghr* KO (Endicott et al., 2021), PTEN overexpressor (Zhang et al., 2023a), and normal animals subjected to calorie restriction (Jafari et al., 2024). This suggests that the activation of CMA is not limited to a single genetic or dietary manipulation, but is a consistent phenomenon across various models of longevity. Near-term work will address the question of whether or not alpelisib treated mice [which are long-lived (Hedges et al., 2023)] also have enhanced CMA, which is highly likely to be the case, given that mice treated with other PIK3CA inhibitors buparlisib and pictilisib have enhanced CMA (Endicott et al., 2020).

Several proteins with certain relevance to aging–MYC, NLRP3, ACLY, and ACSS2 – are degraded by CMA. These proteins are involved in pathways relevant to cancer, metabolism, and proteostasis. The enhanced lifespans of NLRP3 knockout mice (Marín-Aguilar et al., 2020; Navarro-Pando et al., 2021) and MYC hemizygous mice (Hofmann et al., 2015) provide solid evidence of the potential of these proteins as regulators of aging.

While lifespan extending interventions have been shown to enhance CMA, it has never been shown that CMA is required to gain the beneficial effects of lifespan extending interventions. Thus, the relationship between CMA and longevity is only correlational at this stage. Moreover, all of the lifespan extending interventions that enhance CMA are known to affect multiple pathways associated with mammalian aging. Thus, at present, it is not possible to disentangle the role of CMA in longevity from the effects of pathways that work alongside (or cooperate with) CMA.

Furthermore, while many of the proteins degraded by CMA are shown to influence lifespan independently, direct evidence that CMA activation alone extends lifespan through these substrates is lacking. It is unclear whether CMA’s effects on lifespan are entirely due to the degradation of the specific targets named here (MYC, NLRP3, ACLY, and ACSS2) or whether other CMA-sensitive proteins play a role.

Some of the findings linking CMA to longevity might be cell or tissue type specific. Much of the relevant work has focused on the liver, because of the biochemical tractability of this organ. It might be the case that CMA is not affected in the same way across multiple tissues. Even if CMA is uniformly upregulated across tissues in long-lived mice, tissue-specific mechanisms of transcriptional and translation control of protein abundance, along with tissue specific functions of CMA substrates, make it difficult to predict how CMA will affect the biology of individual organs. Thus, extensive tissue specific studies will be required to fully understand the physiological role of CMA in aging and longevity.

### 7.2 Discussion of the aging model

The mechanism through which CMA inhibits oncogenesis and neurodegeneration can be attributed to CMA target proteins. CMA degrades proteins with oncogenic potential like CIP2A (Gomes et al., 2017), MDM2 (Lu et al., 2010), and mutant TP53 (Vakifahmetoglu-Norberg et al., 2013), providing tumor-suppressive functions. Similarly, the degradation of aggregation-prone proteins like APP, MAPT, SNCA (Cuervo et al., 2004), and others provides a clear link between CMA and neurodegeneration. The use of CMA activators (such as the RARA antagonist AR7) to improve proteostasis in models of Alzheimer’s disease provides promising preclinical data that CMA activation could be therapeutically beneficial in neurodegenerative disorders.

LAMP2A knockout mice provide compelling evidence that CMA deficiency directly contributes to disease onset. Mice with liver-specific LAMP2A deletion spontaneously develop liver tumors (Schneider et al., 2015), while neuron-specific LAMP2A knockout mice develop neurodegeneration early in life (Bourdenx et al., 2021). These findings underscore CMA’s protective role against cancer and neurodegenerative disorders.

The primary limitation of the Aging Model is that age-related changes in CMA are highly context dependent, varying widely between cell types, tissue types, and genetic backgrounds. Furthermore, some studies that evaluated changes in CMA with age used animals that were beyond the median lifespan of the stock, raising the possibility that some of the observed changes in CMA or CMA related proteins could be the result of terminal illness, rather than an effect of normal aging. Furthermore, some studies used very old mice from isogenic mouse stocks that are prone to age-related cancers in the same tissue that was examined for changes in CMA. Thus, it might be the case that the results of these studies were influenced by the presence of cancerous cells. Regardless, these findings remain consistent with the idea that loss of CMA is pro-oncogenic, and further underscore the importance of maintaining healthy CMA to protect against cancer.

### 7.3 The future of CMA in multi-omics studies of aging and longevity

Several recent multi-omics studies have shown that there is a poor correlation between mRNA abundances and the abundances of their corresponding proteins in bacteria, yeast, and mammalian cells. This suggests that post-transcriptional mechanisms, including selective translation of mRNAs and selective protein degradation, play very important roles in regulating protein abundance (Vogel and Marcotte, 2012). Studies that have correlated absolute abundances of mRNAs and proteins in bacteria, yeast, and mammals find that about 60% of protein abundance can be explained by mRNA levels (Vogel and Marcotte, 2012). However, when protein and mRNA levels are measured in response to perturbations or stressors, the relative abundance changes in mRNA do not predict the relative abundance changes in proteins (Vogel and Marcotte, 2012).

The poor correlations between mRNA and protein levels are especially relevant to researchers interested in the effects of biological aging on the transcriptome and proteome. Age-associated changes in mRNA either do not correlate or correlate only weakly with age-associated changes in protein in the mouse kidney (Takemon et al., 2021), the mouse heart and skeletal muscle (Han et al., 2021), the mouse liver (Williams et al., 2022), the rat brain and liver (Ori et al., 2015), and human skeletal muscle (Gueugneau et al., 2021). Future multi-omic studies might be able to explain some of the discrepancies between mRNA and protein data by measuring differences in mRNA translation (by ribosome profiling and/or polysome profiling) and by measuring differences in protein degradation (by lysosomal targetomics).

Several recent studies have performed proteomics on isolated lysosomes from mice or cultured cells treated with protease inhibitors, so that lysosomal targets accumulate in the lysosomes without being degraded. These studies have shown that CMA regulates the abundances of specific proteins, which represent a small subset of the proteome (Schneider et al., 2014; Hao et al., 2019; Kacal et al., 2021; Endicott et al., 2022).

Proteins within this CMA-regulated subset of the proteome are often involved in glycolysis (Schneider et al., 2014), translation at the cytoplasmic ribosome (Hao et al., 2019; Kacal et al., 2021; Endicott et al., 2022), and fatty acid metabolism (Schneider et al., 2014; Endicott et al., 2022). Future studies combining lysosomal targetomics with whole-tissue proteomics and transcriptomics could be used to evaluate the hypothesis that protein degradation through CMA will explain some of the discordance between mRNA and protein levels.

### 7.4 The future of CMA as an anti-aging intervention

There are currently no clinically approved treatments for activating CMA therapeutically. A single family of compounds have been patented as CMA activators, i.e., atypical retinoids that antagonize the retinoic acid receptor (Cuervo et al., 2023). This patent has been licensed by Life Biosciences, a Boston-based venture biotech that advertises the goal of developing therapeutics that “can reverse and prevent multiple age-related conditions.”

Retinoic acid (RA) is a derivative of vitamin A that regulates gene expression through retinoic acid receptors (RARs). RARs localize to the nucleus, and act as transcriptional activators when bound to RA (Rhinn and Dollé, 2012). RA plays an essential role in tissue patterning during embryogenesis, and it is an essential regulator of somitogenesis, neural tube development, and limb formation (Rhinn and Dollé, 2012). In adults, RARs play an important role in myelocyte differentiation (Brown, 2023). Acute promyelocytic leukemia can be effectively treated with a high dose of all-trans-retinoic acid (ATRA), which causes the promyelocytic cancer cells to terminally differentiate, thereby exiting the cell cycle (Warrell et al., 1991). Retinoic acid receptor alpha (RARA) is broadly expressed and is actively under investigation as a target for male contraception, due to its indispensable role in spermatogenesis (Noman et al., 2020).

NIH3T3 cells depleted of RARA by shRNA have increased CMA and decreased macroautophagic flux (Anguiano et al., 2013). This observation has led to the development of atypical retinoids that act as small molecule antagonists of RARA (Anguiano et al., 2013). RARA antagonists should not be confused with the subset of atypical retinoids that activate RARA, which are used in cancer research (Altucci et al., 2007).

Anguiano et al. (2013) generated a library of 29 RA-related compounds and screened them for CMA activating activity in NIH3T3 cells, and found three compounds (AR7, GR1, GR2) that activated CMA without inhibiting macroautophagy. Subsequently, AR7 was structurally modified to produce a new generation of compounds, including CA39 and CA77, which are far more potent CMA activators (Gomez-Sintes et al., 2022). In NIH3T3 cells, CA39 and CA77 induce the expression of genes involved in CMA, including LAMP2A and HSC70 (Gomez-Sintes et al., 2022). When these two drugs were administered to mice expressing a fluorescent CMA reporter (Dong et al., 2020), increased CMA activity was observed in several tissues, including the liver, brain, and retina (Gomez-Sintes et al., 2022).

CA77 was administered via intraperitoneal injection for once daily for 1 week to mice with retinal degeneration arising from a missense mutation in *pde6b*. CA77 increased the expression of LAMP2A in the retina and slowed the progression of the retinal degeneration phenotype (Gomez-Sintes et al., 2022). A CMA activating compound of the atypical retinoid variety (whose structure and name were not revealed) was orally administered to old mice, or to hematopoietic stem cells (HSCs) isolated from old mice, and was found to improve several metrics of cell viability and metabolic flux (Dong et al., 2021). Moreover, as mentioned above, CA77.1 significantly delayed neurodegeneration in the PS19 mouse line (Bourdenx et al., 2021).

The initial landmark studies characterizing atypical retinoids as CMA activators have yielded very promising results. However, it is not yet clear whether RARA inhibitors will be beneficial in slowing physiological aging, or if they will be best suited to treating a subset of age-related diseases.

Even though RARA inhibitors are the only class of molecules under development for activating CMA clinically, it is reasonable to hypothesize that drugs that benefit glucose homeostasis and reduce INS spikes (such as acarbose and SGLT2 inhibitors) might already boost CMA. This hypothesis is supported by the findings that CMA is enhanced in mice that are calorie restricted (Jafari et al., 2024), that have reduced circulating INS and IGF1 (such as Snell dwarf, *ghr* KO, and PTEN overexpressor) (Endicott et al., 2021; Zhang et al., 2023a), and mice treated with small molecule inhibitors of class I PI3Ks, which act downstream of the INS receptor (Endicott et al., 2020). If this hypothesis is shown to be correct, then blood-glucose lowering drugs like acarbose and canagliflozin might become favored as CMA activators, since they have a long history of being safe in humans, and they are well-established in their ability to extend mouse lifespan, particularly in males (Harrison et al., 2014; 2019; Jiang et al., 2023; Miller et al., 2024).

## Abbreviations

CMA, chaperone-mediated autophagy; HSC, hematopoietic stem cell; INS, insulin; IGF1, insulin-like growth factor 1; LAMP2A, splice variant A of the LAMP2 gene; mA, microautophagy; MA, macroautophagy.
